# Supplementary figures and images for: Morphological diversity within a core collection of subterranean clover (Trifolium subterraneum L.): Lessons in pasture adaptation from the wild
Source: PLoS One. 2020 Jan 8;15(1):e0223699. doi: 10.1371/journal.pone.0223699 (PMC6949112; doi:10.1371/journal.pone.0223699)

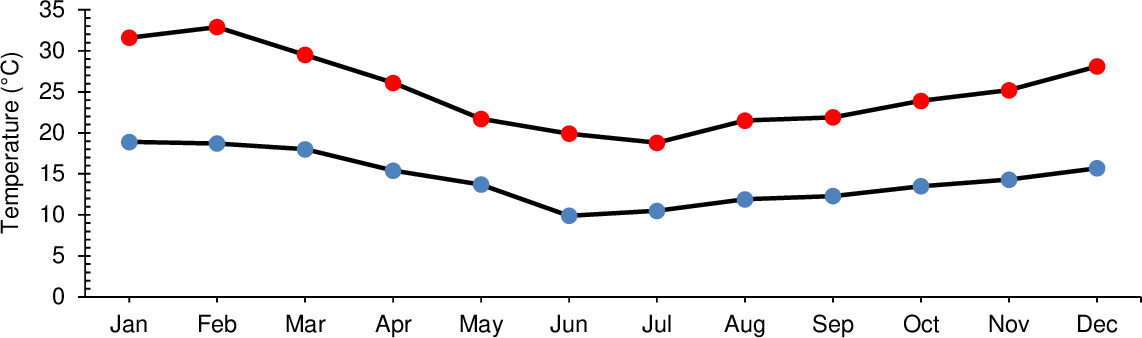

Supplement: S1 Fig — (TIF) [file pone.0223699.s001.tif]

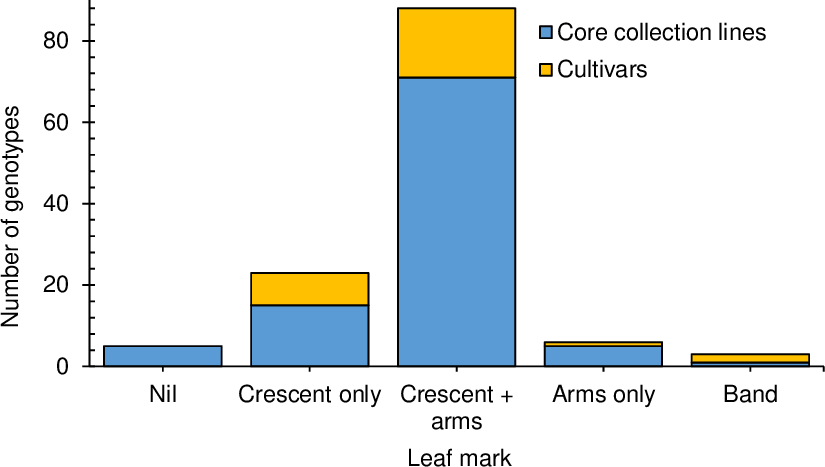

Supplement: S2 Fig — Rating categories are given in S2 Table. (TIF) [file pone.0223699.s002.tif]

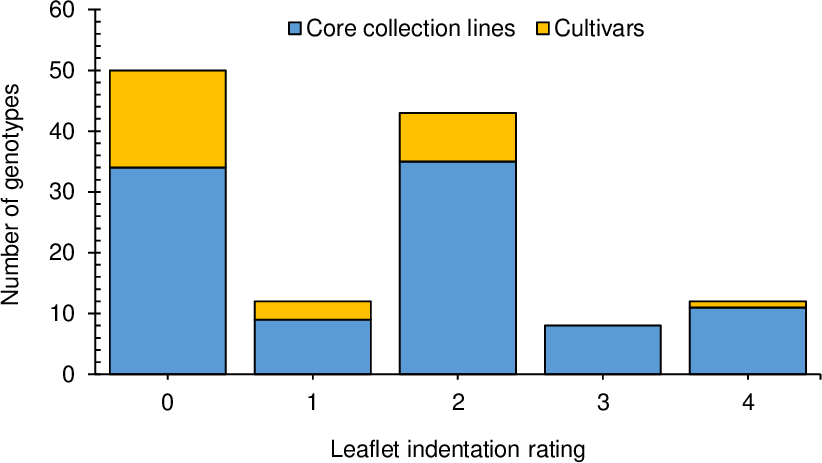

Supplement: S3 Fig — Rating categories are given in S2 Table. (TIF) [file pone.0223699.s003.tif]

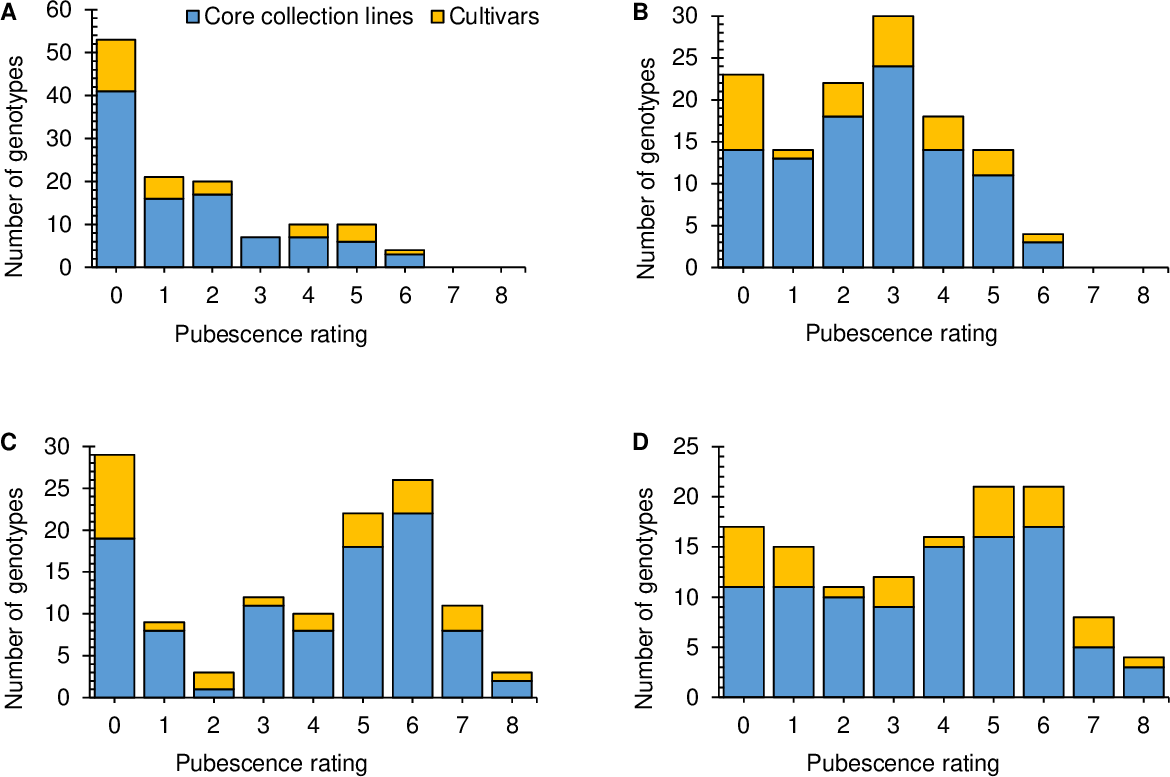

Supplement: S4 Fig — (A) Leaf upper surfaces. (B) Petioles. (C) Stems. (D) Peduncles. Ratings for leaf upper surfaces and petioles were conducted on September 5, while ratings for stems and peduncles were made two weeks after the commencement of flowering. Rating categories are given in S2 Table. (TIF) [file pone.0223699.s004.tif]

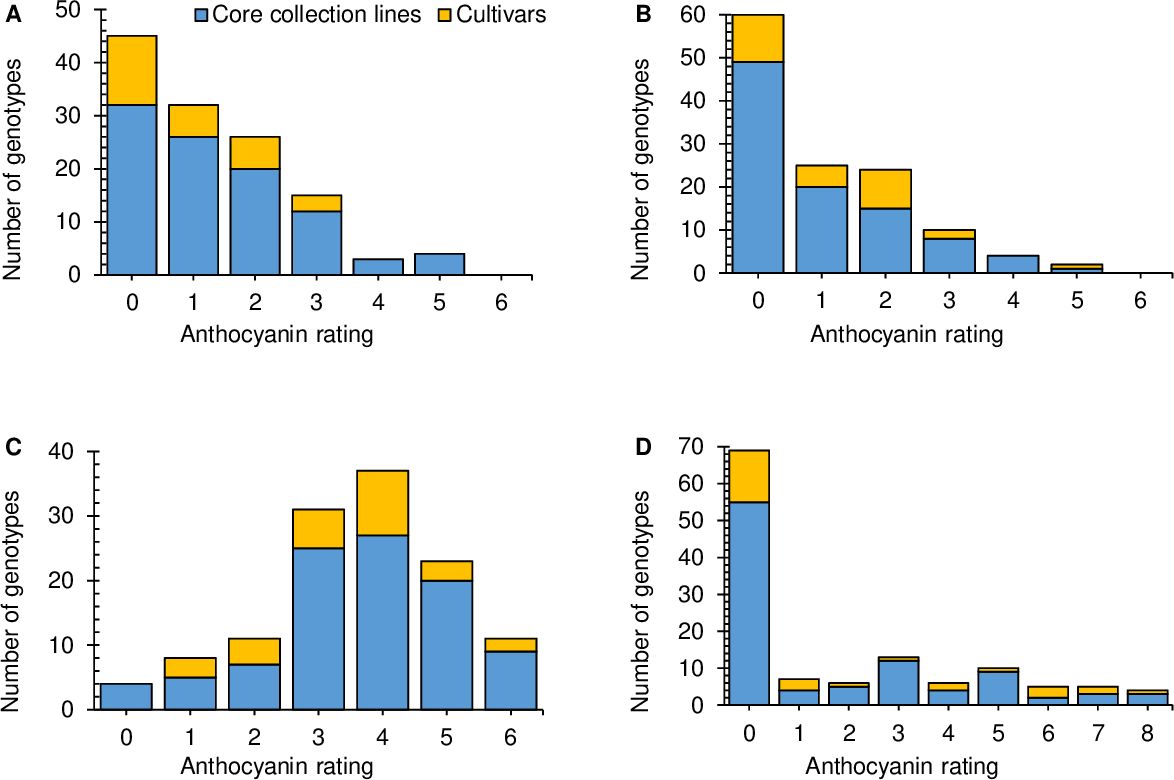

Supplement: S5 Fig — (A) Leaf flecks. (B) Leaf flush. (C) Stipules. (D) Calyx tubes. Ratings for leaf flecks, leaf flush and stipule anthocyanin were conducted on September 5, while ratings for calyx tube pigmentation were made two weeks after the commencement of flowering. Rating categories are given in S2 Table. (TIF) [file pone.0223699.s005.tif]
