# Supplementary material for: Morphological diversity within a core collection of subterranean clover (Trifolium subterraneum L.): Lessons in pasture adaptation from the wild
Source: PLoS One. 2020 Jan 8;15(1):e0223699. doi: 10.1371/journal.pone.0223699 (PMC6949112; doi:10.1371/journal.pone.0223699)
Supplement: Attachment — Submitted filename: Response to reviewers.docx [file pone.0223699.s015.docx]

# Response to reviewers’ comments on paper PONE-D-19-26944 “Morphological diversity within a core collection of subterranean clover (*Trifolium subterraneum* L.): lessons in pasture adaptation from the wild”

In this response, Line numbers refer to those in the original submitted manuscript.

Review 1

**General comments**

The authors present A LOT of data to show that subterranean clover is morphologically highly diverse. This claim is supported by the results. The manuscript is fairly easy to read, even though there are lots of technical details. In my opinion the English is good.

The terms 'diversity' and 'variation' seem to be used interchangeably, please be more clear, also in the hypotheses.

*The term ‘variation’ has been replaced with ‘diversity’ throughout the manuscript for consistency.*

Presentation of the results of the statistical analyses is not always clear.

*Concerns with the presentation of some individual statistical analyses raised by Reviewer 1 have been addressed in the comments below. The other reviewers did not raise any concerns about the statistical analyses.*

Specific comments attached to manuscript

Line 193 and further on: did you account for an edge effect?

*The issue of potential edge effects has been clarified with the additional sentence “This spacing was sufficient to allow plants to grow without competition for light and moisture and prevented edge effects”. It is also clarified in the following sentence referring to additional plants to calibrate shoot dry weight measurements by adding “using the same spacing”.*

Line 238: Explain how are the parameter values determined?

*Determination of plant dry weight has been explained better by referring specifically to the calibration plants used to estimate the regression equation.*

Figures 2-6: some kind of error measure would greatly improve these figures.

*Figures 2-6 are frequency distributions of qualitative, rather than quantitative, traits and consequently, there are no appropriate statistical error measurements that can be displayed. Titles for the vertical axes of each of these graphs have been updated for greater clarity to indicate that data in each case represents numbers of genotypes in each category.*

Figs 3-6 are stacked bar charts, whereas fig2 is a side-by-side bar chart, consistency could be improved.

*Figure 2 has been changed to a stacked bar chart for consistency.*

Fig 5 and 6 lack a legend.

*Legends for figures 5 and 6 have been removed from the caption and placed directly onto the figures for greater clarity.*

Line 348: in addition to the reference please state how you calculated heritability.

*The calculation of heritability has been clarified by the following sentence: “Broad-sense heritability (H^2^) was estimated for each quantitative trait as the percentage of the phenotypic variance attributable to genotypic variance, in accordance with Falconer et al. [38]”.*

Line 355 and table 3: presentation of this result not very clear, all differences were significant, differences between what? Same for line 365 and further on.

*Differences were highly significant for all traits measured among the 125 genotypes at a level of P ≤ 0.001. This is explained in the caption for Table 3. The footnote “ *P ≤ 0.05, **P ≤ 0.01, ***P ≤ 0.001” at the bottom of Table 3 is superfluous and a potential source of confusion for this table and has been removed.*

Flowering time is different among ALL 125 genotypes? Would be more clear if F and p-values would be mentioned.

*A value of P ≤ 0.001 for flowering time is given in Table 3, indicating a significant difference between the 125 genotypes for flowering time. The P value (P ≤ 0.001) has been added to the text in Line 365, as suggested, for greater emphasis.*

Figure 2, legend: 'values are the mean of four replicate single plants' If measurements are done on four out of 97 or 28 plants This should be specified in the methods sections.

*The methodology of measuring flowering time of each genotype on four single plants is explained in detail in the Materials and Methods. The sentence “Values are the mean of four replicate single plants” has been removed from the caption for Figure 2 for simplicity. Information has also been removed from subsequent captions, where it is explained in detail in the Materials and Methods and is not required for interpretation of the figures.*

Table 5: maybe color-code significance for easier reading/interpretation of the table?

*The different levels of significance have been colour-coded for each trait correlation in Table 5, as suggested by the reviewer. The same colour-coding has also been applied to Tables 6-9. The colour scheme can be modified (or deleted) to suit the journal, according to the editor’s suggestions.*

Review 2

**General comments**

The authors present a comprehensive study on a core collection of subterranean clover species consisting of 97 lines, which accounted for approximately 80% of the genetic diversity of the global subterranean clover collection along with 28 Australian cultivars. Assessments were made based on morphological marker traits, agro-morphological traits and phytoestrogen levels in subterranean sp. Trait associations were evaluated based on correlation. It is this reviewer’s opinion that the manuscript presents a comprehensive body of work that will contribute towards developing new cultivars better-suited for establishment in southern Australian agro-climatic conditions. The manuscript is organised in a logical manner, in sound English with only minor typographical errors.

Throughout the manuscript the abbreviation “ssp.” is misrepresented as “spp”, intermittently and requires correction.

*The abbreviation for subspecies has been corrected to “ssp.” throughout the manuscript.*

Specific comments attached to manuscript

Line 150: should read “DNA was extracted from each member of this subset and an 20 additional Australian cultivars. Forty eight SSR primers spread across each of the eight subterranean clover chromosomes….”

*These two sentences have been corrected as advised.*

Line 306: It is best to be more descriptive with the method used. For example the “alcohol” used in this method is ethanol.

*The term “alcohol” has been corrected to “ethanol”.*

While the method for quantifying isoflavones used in this study is valid, the authors should further identify the limitations in using thin layer chromatographic techniques due to the semi quantitative nature and elude to the fact that more precise LC-MS approaches can be useful for absolute quantification of isoflavones. It is logical that the choice of method was due to the high-throughput nature and lower cost associated with TLC, nevertheless I suggest the authors acknowledge that newer technologies can be utilised.

*This point has been addressed by adding the following sentences: “This method was used due to its high-throughput and low cost, while recognising its semi-quantitative nature imposes limitations in precision. This method has also been previously used in similar genetic diversity studies by Nichols et al. [16] and Ghamkhar et al. [31]”.*

The authors observed associations between several morphological markers and environmental factors and suggest that the adaptions are possibly associated with the regulation of genes or interaction between several genes. The authors should also however elude to possibility of epigenetic regulation leading to observed traits.

*We have incorporated the reviewer’s comment on the possible role of epigenetic inheritance by adding the sentence to the Discussion “Epigenetic inheritance may also be important and needs further investigation”. The reference “Quadrana L, Colot V. Plant Transgenerational Epigenetics. Ann. Rev. Genet. 2016; 50: 467-491” has also been added.*

Review 3

**General comments**

The manuscript is very interesting and technically sound. I think it is an important study of morphological diversity of *Trifolium subterraneum* compiling a large data set of several countries. It was possible to link morphological marker traits with environmental factors, being a great contribute to this research subject.

Specific comments attached to manuscript

I suggest an improvement of the graphic quality of figures 4 and 5 (legends…)

*The graphic quality of Figures 3, 4 and 5 have been improved. Legends for Figures 5 and 6 have been placed directly onto the figures for greater clarity.* *Titles for the vertical axes of each graph in Figures 2-6 have been updated to indicate that data in each case represents numbers of genotypes in each category.*

I suggest a revision of typos

*The manuscript has been proofread again and typos have been corrected.*

Line 109 “Chile, Iran Portugal and South” should be replaced by ““Chile, Iran, Portugal and South”.

*This sentence has been corrected.*
