# Supplementary material for: Morphological diversity within a core collection of subterranean clover (Trifolium subterraneum L.): Lessons in pasture adaptation from the wild
Source: PLoS One. 2020 Jan 8;15(1):e0223699. doi: 10.1371/journal.pone.0223699 (PMC6949112; doi:10.1371/journal.pone.0223699)
Supplement: Attachment — Submitted filename: Response to academic editor comments.docx [file pone.0223699.s016.docx]

# Response to Academic editor’s comments on paper PONE-D-19-26944R1 “Morphological diversity within a core collection of subterranean clover (*Trifolium subterraneum* L.): lessons in pasture adaptation from the wild”

I observe that there are 9 tables and 7 figures in the main text. This is too many for a traditional research paper. Please consider replacing some figures or tables in the Supplementary material.

I have reduced the number of figures (four) and tables (two) from the main text and moved them to the Supplementary material, as suggested. This now means the main text has three figures and seven tables. Please advise if this is still too many.

In particular, the original Table 1 is now S2 Table, original Table 2 is now S3 Table, original Fig 3 is now S2 Fig, original Fig 4 is now S3 Fig, original Fig 5 is now S4 Fig and original Fig 6 is now S5 Fig. The text has been modified to take account of these changes.

Improve the quality of the figures: add major and minor marks on the axes, remove or reduce the empty spaces (ex: the empty spaces between vertical bars).

The graphs have been improved, as suggested. Major and minor tick marks have been added to the axes and the spaces between vertical bars in the bar graphs have been markedly reduced.
